# The Persistence of Fluoroquinolone-Resistant *Campylobacter* in Poultry Production

**DOI:** 10.1289/ehp.10050

**Published:** 2007-03-19

**Authors:** Lance B. Price, Leila G. Lackey, Rocio Vailes, Ellen Silbergeld

**Affiliations:** 1 The Johns Hopkins University School of Medicine, Baltimore, Maryland, USA; 2 The Johns Hopkins University, Bloomberg School of Public Health, Baltimore, Maryland, USA

**Keywords:** antibiotic, antimicrobial, *Campylobacter*, chickens, ciprofloxacin, fluoroquinolones, food microbiology, poultry, resistance, veterinary

## Abstract

**Background:**

The use of antibiotics in food animal production has been associated with antibiotic-resistant infections in humans. In 2005, the Food and Drug Administration (FDA) banned fluoroquinolone use in U.S. poultry production in order to reduce the prevalence of fluoroquinolone-resistant *Campylobacter*. Little is known about the potential efficacy of this policy.

**Objectives:**

Our primary objective was to follow temporal changes in the prevalence of fluoroquinolone-resistant *Campylobacter* among poultry products from two conventional producers who announced their cessation of fluoroquinolone use in 2002 (3 years before the FDA’s ban). Our secondary objective was to compare, over time, the prevalence of fluoroquinolone-resistant *Campylobacter* in conventional poultry products to those from producers who claim to use no antibiotics.

**Methods:**

We collected poultry samples from two conventional producers and three antibiotic-free producers over the course of 20 weeks in 2004 (*n* = 198) and 15 weeks in 2006 (*n* = 210). We compared the rates of fluoroquinolone resistance among *Campylobacter* isolates from the different producers.

**Results:**

We found no significant change in the proportion of fluoroquinolone-resistant *Campylobacter* isolates from the two conventional producers over the study period. In addition, *Campylobacter* strains from the two conventional producers were significantly more likely to be fluoroquinolone resistant than those from the antibiotic-free producers.

**Conclusions:**

The results from this study indicate that fluoroquinolone-resistant *Campylobacter* may be persistent contaminants of poultry products even after on-farm fluoroquinolone use has ceased. The FDA’s ban on fluoroquinolones in poultry production may be insufficient to reduce resistant *Campylobacter* in poultry products.

Resistance to antimicrobials is a growing crisis in clinical medicine, and it is generally recognized that misuse and overuse in any sector contributes to this burden. Antimicrobial use in food animal production is an area of concern because the on-farm selection of antimicrobial-resistant zoonotic pathogens can lead to human exposure and infection via various pathways, including meat and poultry products. Fluoroquinolone use in poultry production selects for fluoroquinolone-resistant *Campylobacter* populations and is associated with an increase in fluoroquinolone-resistant *Campylobacter* infections in humans via poultry exposure (Gupta et al. 2004, 2005).

*Campylobacter* is an important foodborne zoonotic pathogen causing enteritis and diarrhea (campylobacteriosis). *Campylobacter* infection is also associated with a number of rare neuropathologic sequelae, including Guillain-Barré syndrome (Hughes et al. 1999). In the United States, *Campylobacter* is the most common cause of bacterial diarrhea, with over a million people estimated to be affected annually [Centers for Disease Control and Prevention (CDC) 2005]. Campylobacteriosis is typically self-limiting, with symptoms rarely lasting more than 10 days (Butzler 2004; CDC 2005); however, it can be fatal in more vulnerable populations (Djuretic et al. 1996; Manfredi et al. 1999; Tee and Mijch 1998).

Indeed, antimicrobial therapy is essential for elderly, pregnant, and immunocompromised patients for whom hydration and electrolyte maintenance may be insufficient (Allos 2001). Until recently, fluoroquinolones were regularly prescribed for those requiring antimicrobial therapy. However, a sharp increase in the prevalence of fluoroquinolone-resistant *Campylobacter,* shown to occur in parallel with the use of fluoroquinolones in U.S. poultry production, has limited fluroquinolones’ effectiveness in the clinical setting (Allos 2001; Collignon 2005; Gupta et al. 2004). Immunocompromised patients with *Campylobacter* bacteremia often require a prolonged course of multiantimicrobial therapy (Tee and Mijch 1998); therefore, the loss of fluoroquinolones as an effective therapeutic has become a threat to these patients.

Based on a risk assessment of the contribution of fluoroquinolone use in poultry production to fluoroquinolone-resistant *Campylobacter* infections in humans, the Food and Drug Administration (FDA) suspended all fluoroquinolone use in poultry production as of 12 September 2005 (FDA 2000). The goal of this policy is to eliminate on-farm selection of fluoroquinolone-resistant *Campylobacter* and thereby reduce human exposure via food to these organisms. However, this policy’s efficacy may be limited by stable reservoirs of fluoroquinolone-resistant *Campylobacter* strains in and around poultry production facilities. These reservoirs can serve to sustain resistant *Campylobacter* in poultry environments, even after the cessation of on-farm fluoroquinolone use (Bull et al. 2006; Moore et al. 2006). Furthermore, some studies indicate that fluoroquinolone-resistant *Campylobacter* isolates may actually be more fit than the wild-type with respect to poultry colonization (Zhang et al. 2006). Therefore, to better assess this policy’s efficacy, it is essential to monitor the prevalence of resistant strains in poultry flocks, production facilities, consumer poultry products, and human infections. If resistant strains continue to persist in spite of the fluoroquinolone ban, it may be necessary to implement other measures in order to reduce fluoroquinolone-resistant *Campylobacter* populations.

Previously, we reported that poultry products from two conventional producers were more likely to be contaminated with fluoroquinolone-resistant *Campylobacter* than products from producers who claimed to use no antibiotics (Price et al. 2005), even though both conventional producers had announced discontinuation of fluoroquinolone use 1 year before the study. Because of the relatively short period of time between this announcement and our analysis, we undertook the current study of products for an additional 3 years (i.e., 4 years beyond the point at which these two companies committed to stop using fluoroquinolones).

## Methods

### Poultry producers

We included products from five different poultry producers in the present study: A) Bell & Evans (Fredericksburg, PA); B) Murray’s (South Fallsburg, NY); C) Eberly (Stevens, PA); D) Perdue (Salisbury, MD); and E) Tyson (Springdale, AR). Producers A–C claim that their chickens are raised without any antibiotics, including fluoroquinolones. We refer to these producers and their products as “antibiotic-free.” Producers D and E do not claim general prohibitions on antibiotics; we refer to these producers and their products as “conventional.” A critical caveat to this designation is that each of the conventional producers announced separately in February 2002 that they had adopted company policies prohibiting the use of fluoroquinolones. In the same announcement, producer D claimed that no fluoroquinolones had been used in the year before the announcement. Finally, all producers except producer C claimed to exclusively slaughter their own flocks in their processing plants. Representatives from producer C acknowledged that “custom flocks,” including those treated with antibiotics, were occasionally processed in their facilities during the study period.

### Sampling and enrichment

We purchased fresh chicken products from grocery stores in the Baltimore, Maryland, area on a weekly basis from 19 January 2004 to 7 June 2004 and from 20 February 2006 to 5 June 2006. Two to three packages from each of the five producers were purchased each time (except when availability was limited). Thighs and legs (bone-in and skin-on) were the default cuts for the study. However, these cuts were not consistently available for all producers; in those cases, we tested alternative cuts, including breasts, quarters, and whole chickens. Packages were refrigerated at 4°C until they were sampled (within 48 hr of purchase). A single piece of chicken was sampled from each package as follows. First, each package was wiped with 70% ethanol and cut open with a new disposable razor blade; the plastic cover was then removed and photocopied for our records. We used sterile forceps to transfer the entire piece of chicken to a stomacher bag containing 200 mL sterile Bolton broth (Oxoid, Hampshire UK) supplemented with laked horse blood (Quad Five, Ryegate, MT); samples were shaken by hand for 2 min, the chicken was removed using forceps, and the bag was sealed 1–2 cm above the top of the broth. Enrichments were incubated at 42°C for 22–26 hr (Hunt 2000; Price et al. 2005).

### Isolation

Ten microliters of the enrichment (~ 10^6^ colony forming units) was streaked onto CCDA (blood-free *Campylobacter* medium; Oxoid) and incubated for 22–26 hr at 42°C. A single typical *Campylobacter* colony was transferred to a fresh CCDA plate and streaked for isolated colonies (this process was repeated once to insure the isolation of a single strain). A single purified colony was then streaked for confluent growth on CCDA and incubated for 22–26 hr. A 10-μL loop-full of cellular material was transferred to *Campylobacter* freezing medium (Hunt 2000), frozen on dry ice, and stored at −80°C.

### DNA isolation

DNA was isolated using a rapid freeze–thaw method. Briefly, one 10-μL loop-full of cellular material was transferred to 150 μL Tris-EDTA in a 200-μL capacity polymerase chain reaction (PCR) tube or 96-well PCR plate. Cellular suspensions were covered and placed in a chilled aluminum block on dry ice for 2 min. Frozen cellular suspensions were then heated in a 95°C aluminum block for 2 min. This process was repeated three times, ending with a final denaturing step of 95°C for 10 min. Cellular debris was pelleted by centrifugation, and 100 μL supernatant was transferred to a fresh PCR tube or 96-well PCR plate.

### Species confirmation

Presumptive *Campylobacter* isolates were confirmed and the species identified using a PCR amplification/ restriction digest described previously (Engvall et al. 2002). Briefly, THERM1 and THERM4 PCR primers were used to amplify a region of DNA specific to thermophilic members of the genus *Campylobacter*. This PCR product was then digested in two separate reactions using the restriction endo-nucleases, *Alu*I and *Tsp*509I (New England Biolabs, Ipswich, MA). The restriction patterns produced from this digestion are distinctive among the thermophilic *Campylobacter* species (Engvall et al. 2002).

### Susceptibility

Susceptibility to fluoroquinolone was determined using standard Clinical and Laboratory Standards Institute methods and *Campylobacter*-specific methods described previously by McDermott and Walker (2003). Briefly, *Campylobacter* isolates were grown overnight on CCDA under microaerophilic conditions. Colonies were suspended to approximately 0.5 McFarland standard in Mueller-Hinton broth and inoculated onto Mueller-Hinton agar supplemented with 5% sheep blood and ciprofloxacin (USBiological, Swampscott, MA) at concentrations of 0.12–32 μg/mL. Plates were grown 22–26 hr at 42°C under microaerophilic conditions. The reference strain used was *Campylobacter jejuni* (ATCC 33560; American Type Culture Collection, Manassas, VA). Strains were designated resistant if their minimal inhibitory concentration was ≥ 4 μg/mL.

### Statistical analysis

We performed statistical analyses using Stata 8.0 (StataCorp, College Station, TX). Chi-square analysis was used to compare the proportions of samples testing positive for *Campylobacter* and those positive for *Campylobacter* resistant to fluoroquinolones. Relative proportions with corresponding 95% confidence intervals (CIs) were computed for all pair-wise comparisons of producers. We detected no fluoroquinolone-resistant *Campylobacter* isolates from two producers in 2006 (Table 1); zeros were replaced with ones for relative proportion calculations involving these producers. We used univariate analysis to examine the association between species and fluoroquinolone resistance.

## Results

### Fluoroquinolone resistance

Overall, 13% of *Campylobacter* isolates were resistant to fluoroquinolones in 2004 and 21% in 2006 (a non-significant increase; *p* = 0.06) (Table 1). The proportion of *Campylobacter* isolates resistant to fluoroquinolones did not change significantly between the two test periods for any particular producer (Table 1). The proportion of resistant isolates from the two conventional producers was consistent with those collected in 2003 (Price et al. 2005).

Pair-wise comparisons revealed significant differences in the proportion of fluoroquinolone-resistant *Campylobacter* among the different producers. Without exception, *Campylobacter* from conventional products were more likely to be fluoroquinolone resistant than *Campylobacter* isolated from antibiotic-free products (Table 2). Fluoroquinolone resistance was significantly more prevalent among isolates from conventional products compared with antibiotic-free products (Table 2). These data were consistent with previous product surveys (Cui et al. 2005; Price et al. 2005), as well as with an on-farm study that showed conventionally raised poultry are more likely to be colonized with fluoroquinolone-resistant *Campylobacter* compared with those raised under the U.S. Department of Agriculture (USDA) organic label guidelines (Luangtongkum et al. 2006).

### Campylobacter *contamination.*

*Campylobacter* (undifferentiated by fluoroquinolone resistance) was detected on 77% and 84% of all the chicken products tested in 2004 and 2006, respectively (Table 1), again consistent with previous studies (Cui et al. 2005; Price et al. 2005). Among the five producers, only producer B (antibiotic-free) was significantly more contaminated in 2006 than in 2004 (*p* = 0.006). The reason for this increase is not known, but the increase may reflect changes in production methods that are beyond the scope of this article.

In our pair-wise analysis, significant differences in the prevalence of *Campylobacter* contamination were shown both among the three antibiotic-free producers and between the two conventional producers. We also found significant differences in the prevalence of *Campylobacter* contamination between specific antibiotic-free and conventional producers, but there was no overall difference between the two groups (conventional vs. antibiotic-free) in either year (Table 3).

Of the isolates, 92% were identified as either *Campylobacter coli* (36%) or *C. jejuni* (56%). One isolate was identified as being *Campylobacter lari*, and the remaining isolates were identified as *Campylobacter* spp., based on standard phenotypic analysis. We found no significant difference in the prevalence of fluoroquinolone resistance between the *C. coli, C. jejuni, or Campylobacter* spp. collected in this study (*C. lari* was too rare to contribute significantly to this assessment).

## Discussion

This is the first published study reporting the temporal trends in fluoroquinolone-resistant *Campylobacter* on poultry products from two major U.S. broiler producers after they voluntarily ceased using fluoroquinolones for broiler production. The results of this study indicate that fluoroquinolone-resistant *Campylobacter* may be persistent contaminants of poultry products for years after on-farm fluoroquinolone use has ended.

### Sustained resistance

Poor hygiene practices and insufficient biosecurity measures may play critical roles in sustaining fluoroquinolone-resistant *Campylobacter* populations (Moore et al. 2006; Newell and Fearnley 2003). In the United States, protocols for cleaning broiler chicken houses range from removing the upper layer of litter between every flock to reusing litter for multiple flocks before removal (Morison C, personal communication). Complete *Campylobacter* decontamination is probably rare under any standard practice, and contaminated litter can be a significant source of *Campylobacter* carryover and colonization in poultry houses (Petersen and Wedderkopp 2001). *Campylobacter* in poultry house water distribution systems is another potential reservoir of resistant strains. Although individual *Campylobacter* cells are sensitive to many common disinfectants, they can form disinfectant-resistant biofilms in the water distribution systems of poultry houses (Trachoo and Frank 2002; Trachoo et al. 2002). *Campylobacter* can also reside in protozoa that contaminate water distribution systems, thereby increasing their resistance to chemical disinfectants (Snelling et al. 2005).

Colonization with fluoroquinolone-resistant *Campylobacter* is not limited to *Campylobacter* sources within the broiler facility; the immediate external environment has also been shown to be an important source of *Campylobacter* for colonization. Once a flock becomes colonized with fluoroquinolone-resistant *Campylobacter*, these resistant organisms can be pumped into the environment via tunnel ventilation systems. *Campylobacter* has been detected in the air up to 30 m downwind of facilities housing colonized flocks (Bull et al. 2006). In addition, wild birds and surface waters can also become colonized or contaminated with fluoroquinolone-resistant *Campylobacter*, thereby becoming reservoirs for subsequent flocks (Bull et al. 2006; Chuma et al. 2000; Waldenstrom et al. 2005). Finally, recent studies have demonstrated that houseflies can carry *Campylobacter* and that these flies are undeterred by conventional biosecurity measures, with as many as 30,000 entering a facility during a single flock rotation (Hald et al. 2004). The combination of environmental, animal, and insect reservoirs and potential carriers provide significant challenges to poultry producers who wish to eliminate the fluoroquinolone-resistant *Campylobacter* colonizing their flocks.

The continued presence of fluoroquinolone-resistant *Campylobacter* on poultry products may be a result of more than contamination in and around farms. Controlled physiology experiments indicate that fluoroquinolone-resistant strains may be more fit than wild-type *Campylobacter* in their ability to both colonize and persist in the gut of chickens (Zhang et al. 2006). If these findings hold true in the setting of real-world poultry facilities, merely removing the fluoroquinolones from production may be insufficient to reduce the prevalence of resistant strains.

Although the present study does not include samples from before the voluntary cessation of fluoroquinolone use by two conventional producers, it clearly shows that the prevalence of fluoroquinolone-resistant *Campylobacter* is not decreasing on their products in the years following cessation and has not decreased to the level found on the products of antibiotic-free producers who claim no history of fluoroquinolone use. On the other hand, this study also shows that the resistance is not increasing significantly. In Spain, where fluoroquinolones were used heavily in poultry production, approximately 99% of poultry-associated *Campylobacter* isolates were fluoroquinolone resistant in the late 1990s (Garau et al. 1999; Saenz et al. 2000). Compared with this, holding the proportion of *Campylobacter* resistant to fluoroquinolones < 50% may be considered, by some, to be a victory.

### Antibiotic-free versus conventional products

Consistent with our previous study (Price et al. 2005), *Campylobacter* isolates from antibiotic-free products were significantly less likely to be fluoroquinolone resistant than those from conventional producers. The substantial, although not statistically significant, increase from 2004 to 2006 in fluoroquinolone-resistant strains among *Campylobacter* isolated from the antibiotic-free producer C may be due to cross-contamination from processing equipment previously used to slaughter conventional flocks. Producer C was the only antibiotic-free producer that processed both antibiotic-free and conventional flocks in their processing facilities during the time of the study.

Such cross-contamination may take place in conventional processing facilities as well. For example, it is feasible that some growers who raise chickens under contract with conventional producers have no history of on-farm fluoroquinolone use. These growers may raise flocks that become cross-contaminated by slaughter equipment in conventional slaughter facilities. Previous studies have shown that cross-contamination is a regular occurrence in processing plants (Newell et al. 2001). However, it should be emphasized that antibiotic use on the farm is generally dictated by the producers who control the processing plants and with whom growers contract.

### Campylobacter *contamination.*

The prevalence of *Campylobacter* (susceptible and resistant) in poultry products varied significantly among producers; however, there was no consistent pattern with regard to antibiotic-use group. These findings are consistent with those from Denmark, where *Campylobacter* contamination on poultry products did not change significantly after antimicrobials were removed as feed additives (Evans and Wegener 2003). On-farm and processing plant practices, such as between-flock cleaning and decontamination of slaughter equipment, likely outweigh any impact from antimicrobial use on general *Campylobacter* contamination.

### Limitations of the study

The present study had four primary limitations. First, the study was limited in geographic region; however, despite this limitation, the results are probably generalizable because integrator-defined production methods vary little from region to region. Moreover, broiler production has become regionally concentrated in the southeastern United States, and products from this region are distributed widely throughout the United States. Second, the study was limited in the number of producers included in the study. We focused on conventional producers D and E because they announced that they had ceased using fluoroquinolones in 2002. The three antibiotic-free producers were the only three with branded products consistently available in the Baltimore area. Third, we could not test the same cut each time from each producer. Thighs and legs (bone-in, skin-on) were the default cuts for the study; however, because these cuts were not consistently available for some producers, alternative cuts were tested occasionally. However, because products were tested for the presence or absence of *Campylobacter* rather than by quantifying colony forming units, the choice of cut probably had little, if any, impact on the outcome of the study. Finally, actual use of fluoroquinolones during the test period could not be determined because information on drug use in food animals is considered proprietary and not subject to mandatory disclosure to regulatory bodies (National Research Council 1999).

### Public health implications

Fluoroquinolone-resistant *Campylobacter* strains pose a significant public health threat in the United States. In response to growing concerns over the contribution of agricultural antimicrobial use to resistant human infections, the FDA banned the use of fluoroquinolones in U.S. poultry production (FDA 2000). The results from the present study indicate that fluoroquinolone-resistant *Campylobacter* may be persistent contaminants of poultry products even after on-farm fluoroquinolone use has ceased. Thus, the FDA’s policy alone may be insufficient to reduce consumer exposures to fluoroquinolone-resistant *Campylobacter*. Without additional interventions, fluoroquinolone-resistant *Campylobacter* may continue to be a public health burden for years after the FDA’s ban.

## Figures and Tables

**Table 1:** Prevalence of *Campylobacter* and fluoroquinolone-resistant *Campylobacter* among producers in 2004 and 2006.

	2004			2006		
Producer	No. of samples	Percent (no.) *Campylobacter*^a^	Percent (no.) FQ-resistant *Campylobacter*^b^	No. of samples	Percent (no.) *Campylobacter*^a^	Percent (no.) FQ-resistant *Campylobacter*^b^
Antibiotic-free						
A	40	67.5 (27)	3.7 (1)	45	66.7 (30)	0.0 (0)
B	38	63.2 (24)	4.2 (1)	33	90.9 (30)^c^	0.0 (0)
C	40	92.5 (37)	2.7 (1)	42	95.2 (40)	15.0 (6)
Conventional						
D	40	62.5 (25)	24.0 (6)	45	77.8 (35)	37.1 (13)
E	40	97.5 (39)	28.2 (11)	45	93.3 (42)	42.9 (18)
Total	198	76.8 (152)	13.2 (20)	210	84.3 (177)	20.9 (37)

FQ, fluoroquinolone.

a Percentage of samples contaminated with *Campylobacter* (susceptible or resistant).

b Percentage of *Campylobacter* isolates resistant to fluoroquinolone.

c Significant increase over the 2004 proportion (*p* < 0.05).

**Table 2:** Relative proportions of fluoroquinolone-resistant *Campylobacter* among producers.

		2004		2006	
Reference producer	Comparison producer	Relative proportion resistant (95% CI)	*p-*Value	Relative proportion resistant (95% CI)	*p-*Value
A^a^	B	1.1 (0.1–17.0)	0.932	> 1.0 (0.1–15.3)	1.000
	C	0.7 (0.0–11.2)	0.820	> 4.5 (0.6–35.4)	0.107
	D	6.5 (0.8–50.1)	0.032	> 11.1 (1.5–80.3)	0.001
	E	7.6 (1.0–55.6)	0.011	> 12.9 (1.8–91.1)	0.000
B^a^	C	0.6 (0.0–9.9)	0.754	> 4.5 (0.6–35.4)	0.107
	D	5.8 (0.7–44.4)	0.047	> 11.1 (1.5–80.3)	0.001
	E	6.8 (0.9–49.2)	0.018	> 12.9 (1.8–91.1)	0.000
C	D	8.9 (1.1–69.3)	0.009	2.5 (1.1–5.8)	0.028
	E	10.4 (1.4–76.9)	0.002	2.9 (1.3–6.5)	0.006
D	E	1.2 (0.5–2.8)	0.710	1.2 (0.7–2.0)	0.611
Antibiotic-free	Conventional	7.8 (2.4–25.5)	0.000	5.0 (2.5–10.3)	0.000

The relative proportion resistant is the proportion of fluoroquinolone-resistant *Campylobacter* from the comparison producer divided by the proportion of fluoroquinolone-resistant *Campylobacter* from the reference producer. Each producer was compared with every other producer in a pair-wise fashion.

a Zero counts were replaced with 1 in order to estimate relative proportions; “>” indicates that the replacement of zero values results in an underestimate of the actual relative proportions.

**Table 3:** Relative proportions of *Campylobacter* contamination (susceptible and resistant) among producers.

		2004		2006	
Reference producer	Comparison producer	Relative proportion contaminated (95% CI)	*p-*Value	Relative proportion contaminated (95% CI)	*p-*Value
A	B	0.9 (0.7–1.3)	0.687	1.4 (1.1–1.7)	0.012
	C	1.4 (1.1–1.7)	0.005	1.4 (1.1–1.8)	0.001
	D	0.9 (0.7–1.3)	0.639	1.2 (0.9–1.5)	0.239
	E	1.4 (1.2–1.8)	0.000	1.4 (1.1–1.7)	0.002
B	C	1.5 (1.1–1.9)	0.002	1.0 (0.9–1.2)	0.456
	D	1.0 (0.7–1.4)	0.952	0.9 (0.7–1.0)	0.124
	E	1.5 (0.2–0.5)	0.000	1.0 (0.9–1.2)	0.691
C	D	0.7 (0.5–0.9)	0.001	0.8 (0.7–1.0)	0.018
	E	1.1 (1.0–1.2)	0.305	1.0 (0.9–1.1)	0.703
D	E	1.6 (1.2–2.0)	0.000	1.2 (1.0–1.4)	0.036
Antibiotic-free	Conventional	1.1 (0.9–1.2)	0.375	1.0 (0.9–1.2)	0.662

The relative proportion contaminated is the proportion of products contaminated with *Campylobacter* from the comparison producer divided by the proportion of products contaminated with *Campylobacter* from the reference producer. Each producer was compared with every other producer in a pair-wise fashion.
